# Evaluation of the Effectiveness of the Friends for Life Program on Children’s Anxiety and Depression

**Published:** 2017-10

**Authors:** Fatemeh Moharreri, Aazam-Sadat Heydari Yazdi

**Affiliations:** Psychiatry and Behavioral Sciences Research Center, Mashhad University of Medical Sciences, Mashhad, Iran.

**Keywords:** *Anxiety*, *Depression*, *Children*, *Friends*

## Abstract

**Objective:** Anxiety disorders and depression during childhood and adolescence are among highly prevalent serious mental health problems, which lead to reduced performance in children and can also negatively affect children’s emotional and social long-term development. This study, which was conducted in Mashhad in 2015, aimed at evaluating the effectiveness of the Friends for Life cognitive-behavioral program in reducing the symptoms of anxiety and depression in children.

**Method:** In this controlled clinical trial, 248 male students aged 10 were screened for mild to moderate symptoms of anxiety and depression using the Revised Children’s Manifest Anxiety (RCMA) and the Children’s Depression Inventory (CDI). Of the participants, 40 students met the inclusion criteria.

The demographic questionnaire, the Strengths and Difficulties Questionnaire (SDQ), and the Depression-Anxiety-Stress Scale (DASS) were filled out by parents. The children in the experimental group received the Friends for Life cognitive-behavioral training program for eight 1-hour weekly sessions. RCMA, CDI, SDQ, and DASS were filled out again by both groups at the end of the sessions and 3 months later. To evaluate comparability between the 2 groups, Mann-Whitney test was used for qualitative variables and paired t test and repeated measure for quantitative variables with normal distribution. Statistical analysis was performed using SPSS-16. All statistical references were made at □=0.05.

**Results:** Children’s depression and manifest anxiety scores were not significantly different in the 2 groups before the intervention; however, their changes immediately after intervention and at the 3- month follow-up were significant (p<0.001). Moreover, hyperactivity (p = 0.039), peer problems (p = 0.011), and parental depression (p = 0.015) scores significantly changed in both groups over time.

**Conclusion:** Implementation of Friends for Life program is effective in prevention and treatment of the symptoms of anxiety and depression in children.

Anxiety disorders (1) and depression (2) during childhood and adolescence are among highly prevalent serious mental health problems. Life time prevalence of any anxiety disorders in children and adolescents is between 15% and 32% (3). Anxiety and depression symptoms decrease children’s performance and cause poor social relations, academic performance, and low self-esteem (4). Such disorders can also negatively affect children’s social and emotional long-term development. For example, childhood anxiety and depression are important predictors of psychopathology in adulthood (5, 6). When left untreated, they can increase the risk of developing other psychological disorders (7). 

Therefore, primary prevention of such disorders is of prime importance, which can protect individual interests concerning children’s present and future conditions and reduce the costs associated with such disorders in the society (4).

In a randomized intervention study, Barrett & Turner revealed that the Friends for Life cognitive-behavioral intervention, based on the Coping Cat program designed by Phillip Kendall in 1994, could reduce anxiety symptoms. They recommended that universal interventions be considered at schools accordingly (5).

Friends for Life is a program that can be used for prevention and treatment of anxiety and depression in "Friends for Life" is a program that can be used for prevention and treatment of anxiety and depression in children. This cognitive-behavioral program trains children and empowers them to more effectively cope with feelings of anxiety and depression and learn relaxation, emotional flexibility, problem solving ability, and self-efficacy techniques (8-13).

The only study conducted in Iran was a research entitled, “A Parent-only Group Cognitive Behavioral Intervention for Children with Anxiety Disorders: A Control Group Study”. In this study, which was conducted by Shahrivar et all in 2009, no significant change was found in the total score of the Children’s Manifest Anxiety Scale. Children’s anxiety behaviors, which were based on the emotional symptoms subscale score of strengths and difficulties questionnaire and were scored by parents, significantly decreased. Another important finding of this study was that although parental anxiety scores in the depression anxiety stress scale did not change, parental depression scores significantly decreased. Moreover, the total score of the depression anxiety stress scale decreased, which was not statistically significant (14).

Friends for Life program has been recommended by the World Health Organization (WHO) to prevent the development of anxiety disorders in children (15). In view of the prevalence, severity and economic burden, improving the mental health of children is recognized as a global priority with emphasis on the development of prevention programs. Schools have been advocated as convenient locations to deliver preventive mental health interventions, and they have an important role in helping to develop emotional resilience. While school‐based mental health prevention programs have documented improvements in psychological functioning in other countries (16, 17), little is known about their impact on reducing the symptoms of depression and anxiety in children in Iran. Moreover, differences in school systems, cultural values, children’s racial, social, and economic characteristics as well as participants’ continuity of the treatment program, level of participation, and compliance affect the effectiveness of the program in different countries. In addition, although this program has been proved effective in other countries, its effect size in various studies has ranged from 0.06 to 2.76 (18), and its effect size has not been evaluated in Iran. Therefore, this study aims at evaluating the effectiveness of the Persian version of the Friends for Life program in reducing the symptoms of depression and anxiety in 9 to 12 year-old children in Mashhad in 2015.

## Materials and Methods

This was a controlled clinical trial that evaluated the effectiveness of the Friends for Life cognitive-behavioral program in reducing the symptoms of anxiety and depression in children. The trial was registered at the Iranian registry of clinical trials (www.irct.ir; registration number: IRCT201506215280N18). The trial protocol was approved by ethics committee of Mashhad University of Medical Sciences in April 11, 2015. 

*Participants* *:*

In this study, after negotiation with the principal of school “A”, all students (248 male students aged 10) were screened for mild to moderate anxiety and depression using the Revised Children’s Manifest Anxiety (RCMA) and the Children’s Depression Inventory (CDI), and 40 students met the following inclusion criteria:

1) Children with mild to moderate symptoms of anxiety and depression based on RCMA>17 and CDI = 9-35.

2) Absence of anxiety or depressive disorders demanding pharmacological treatment (Based on a clinical interview, which was done by a child and adolescent psychiatrist based on DSM-V criteria).

3) Absence of other psychiatric disorders demanding pharmacological treatment (Based on a clinical interview, which was done by a child and adolescent psychiatrist based on DSM-V criteria).

*Exclusion criteria were as follow* *:*

1) Missing 2 training sessions or more

2) Recent experience of an acute stressful event

*Procedures* *:*

Parents of the children, who met the inclusion criteria, were invited. After the study method and a summary of the Friends for Life prevention program were explained to the parents, informed consent was obtained from those parents who wanted their children to participate in the study. Children were informed that they were free to withdraw from the trial at any time without any adverse effect on their relationship with their health care provider. To rule out other psychiatric disorders, the children underwent clinical interviews by a child and adolescent psychiatrist based on DSM-IV TR criteria. Then, demographic questionnaire, the Strengths and Difficulties Questionnaire (SDQ), and the Depression-Anxiety-Stress Scale (DASS) were filled out by parents. After that, the children were randomly placed into 2 groups (experimental and control groups) by using the table of random numbers. 

The children in the experimental group received the Friends for Life cognitive-behavioral training program for eight 1-hour weekly sessions by a child and adolescent psychiatrist. Two training sessions were also held for parents after the fourth session and at the end of the sessions, and the control group was put on a waiting list. After establishing initial connections with children and group members’ familiarity with each other, the children were trained during the group training sessions in physiological, cognitive, and behavioral aspects of anxiety, relaxation techniques, identification of negative thoughts and how to replace them with positive thoughts, problem solving ability, planning, and positive reinforcement (Table 1).

At the end of the sessions and 3 months later, the RCMA and CDI were filled out by children and the SDQ and DASS were filled out by parents in both groups.

After data collection, the Friends for Life training sessions were held for the children of the control group by a child and adolescent psychiatrist.

*Measurements*

Revised Children's Manifest Anxiety (RCMA): This self-report questionnaire consists of 28 items assessing a child's chronic or trait anxiety and 9 items assessing social desirability or potential lying. The RCMA has achieved a high internal consistency and moderate test-retest reliability (19-21). Moreover, its validity was reported to be 0.67 in Iran (22).

Children's Depression Inventory (CDI): This self-report inventory has 27 items on the cognitive, affective, and behavioral signs of depression. The scale has high internal consistency and moderate test-retest reliability (23, 24). Moreover, the test-retest reliability was reported to be 0.82 in Iran (25).

Strengths and Difficulties Questionnaire (SDQ): This questionnaire is a parent report of psychopathology in children and adolescents. The scale consists of 25 items, which generates 5 subscale scores (emotional symptoms, conduct problems, inattention/hyperactivity, peer problems, and prosocial behavior) and a total difficulties score. SDQ has adequate internal consistency (α = 0.37) and good test-retest reliability (r = 0.62) (26-28). The validity and reliability of SDQ has been confirmed in community samples of Iranian children and adolescents (29). Depression-Anxiety-Stress Scale (DASS): This is a 42 item self-report instrument designed to measure the negative emotional states of depression, anxiety, and stress. The DASS was shown to possess satisfactory psychometric properties (30-32).

*Statistical Analysis*

To evaluate comparability between the 2 groups, Mann-Whitney test was used for qualitative variables and paired t test and repeated measure for quantitative variables with normal distribution. Statistical analysis was performed using SPSS-16. All statistical references were made at P = 0.05.

## Results

In this study, all students of school “A” (248 male students, mean age 10) were screened for mild to moderate symptoms of anxiety and depression using the Revised Children’s Manifest Anxiety (RCMA) and the Children’s Depression Inventory (CDI). Of the participants, 40 students met the inclusion criteria and were randomly placed into two 20-member groups (experimental and control groups). Of the experimental group, 2 members were excluded from the study: one due to non-participation in training classes after the third session, and the other due to the severity of depression symptoms and the need for medical treatment because of parental divorce and environmental stressors. 

**Table 1:** Outline of 'Friends for life' (based on Barrett PM (9))

**Session ** **Number**	**Content of Session - Major Learning Objectives**
**Session 1**	Rapport building and introduction of group participants Establishing group guidelines Normalization of anxiety and individual differences in anxiety reactions Psycho-education regarding identification of various emotions Introduce the relationship between thoughts and feelings
**Session 2**	**F**: Feelings (Identifying body signs of anxiety) **R**: Remember to relax. Have a quiet time. (Relaxation activities and identification of pleasant or distracting activities to do when feeling worried or sad)
**Session 3**	I: I can do it! I can try my best! (Identifying self-talk, introducing helpful green thoughts and unhelpful red thoughts)
**Session 4**	Attention training (looking for positive aspects in difficult situations) Challenging unhelpful red thoughts **E**: Explore solutions and Coping Step Plans (introducing coping step plans/graded exposure to fear hierarchies, setting goals and breaking problems into small steps)
**Session 5**	Problem-solving skills (6-Stage Problem-Solving Plan) Coping Role models Social support plans
**Session 6**	**N**: Now reward yourself! You've done your best!
**Session 7**	**D**: Don't forget to practice (practicing the FRIENDS skills) **S**: Smile! Stay calm for life! (Reflect on ways to cope in difficult situations)
**Session 8**	Generalizing skills of FRIENDS to various difficult situations Teaching others how to use the FRIENDS coping skills

**Table 2:** Distribution of demographic items in the study groups

**Demographic items**		**Study groups**		**Mann-** **Whitney ** **test**
		**experimental** **frequency (percent)**	**control** **Frequency (percent)**	
**Father's Education**	Diploma	6 (33.3) 11 (61.1) 1 (5.6) 18 (100.0) 8 (44.4) 9 (50.0) 1 (5.6) 18 (100.0) 8 (44.4) 9 (50.0) 1 (5.6) 0 (0.0) 18 (100.0) 3 (16.7) 11 (61.1) 4 (22.2) 0 (0.0) 18 (100.0)	7 (41.2) 7 (41.2) 3 (17.6) 17 (100.0) 8 (47.1) 9 (52.9) 0 (0.0) 17 (100.0) 8 (47.1) 7 (41.2) 1 (5.9) 1 (5.9) 17 (100.0) 3 (17.6) 11 (64.7) 2 (11.8) 1 (5.9) 17(100.0)	0.987
	BS & MS			
	PHD			
	Total			
**Mother's ** **Education**	Diploma			0.782
	BS & MS			
	PHD			
	Total			
**Birth rate**	1			0.961
	2			
	3			
	6			
	Total			
**Number of ** **offspring**	1			0.883
	2			
	3			
	6			
	Total			

**Table 3:** Children’s Depression test score at before, immediately and 3 months after intervention by the control and intervention Groups

	**Study Groups**		**Independent T Test**	
**Children’s Depression**	Experimental	Control		
	Mean± SD	Mean± SD		
**Before Intervention**	6.85± 23.89	8.46± 21.56	0.440	
**Immediately after Intervention**	8.60 ± 11.11	7.48± 22.12	<0.001	
**3 Months after Intervention**	6.94 ± 8.67	8.175 ± 21.19	<0.001	
**Repeated measure test**	<0.001	0.901		

**Table 4:** Children’s Manifest Anxiety test score at before, immediately and 3 months after intervention by the control and intervention Groups

Children’s Manifest Anxiety	**Study Groups**		**Independent T Test**	**Effect Size**
	Experimental	Control		
	Mean± SD	Mean± SD		
**Before Intervention**	2.5 ± 19.61	2.36 ± 19.5	0.864	0.04
**Immediately after Intervention**	5.75 ± 7.0	6.78 ± 18.12	<0.001	1.76
**3 Months after Intervention**	5.19 ± 4.78	5.73± 17.88	<0.001	2.39
**Repeated measure test**	<0.001	0.417		

**Table 5:** Total score of Strengths and Difficulties at before, immediately and 3 months after intervention by the control and intervention Groups

**Strengths and Difficulties**	**Study Groups**			**Effect Size**
	Experimental		Control	
	Mean± SD		Mean± SD	
**Before Intervention**	8.35 ± 13.58		6.99± 12.83	0.09
**Immediately after Intervention**	7.17 ± 12.25		5.37 ± 14.17	0.3
**3 Months after Intervention**	7.03 ± 10.25		5.97± 11.0	0.11
**Repeated Measures Design Test Results**	Time effect	0.004=P	6.29=F	
	Group effect	0.811=P	0.59=F	
	Interaction effect	0.292=P	1.26=F	

**Table 6:** Distribution of Parental Depression-Anxiety-Stress Scale Score at before, immediately and 3 months after intervention by the control and intervention Groups

**Parental Depression-** **Anxiety-Stress**	**Study Groups**			**Effect Size**
	Experimental		Control	
	Mean± SD		Mean± SD	
**Before Intervention**	18.17 ± 17.63		17.0 ± 11.74	0.07
**Immediately after ** **Intervention**	13.67 ± 12.50		17.83 ± 13.03	0.32
**3 Months after ** **Intervention**	8.67 ± 9.09		13.50 ± 10.48	0.49
**Repeated Measures ** **Design Test Results**	Time Effect	P=0.053	F=3.56	
	Group Effect	P=0.549	F=0.37	
	Interaction Effect	P=0.402	F=0.858	

Of the control group, 3 members were excluded from the study because of parents’ non-cooperation in completing the follow-up questionnaires. Therefore, a total of 35 individuals participated in this study, of whom 18 and 17 were placed into the intervention and control groups, respectively.

The result of the Mann-Whitney test revealed that the distribution of father’s education, mother’s education, birth rate, and number of offspring was not significantly different in the study groups.

As tables 3 and 4 demonstrate, children’s manifest anxiety and depression scores before intervention were not significantly different in in the two 2 groups; however, their changes immediately after the intervention and during at the 3- months of follow-up were significantly different in in the two 2 groups. Moreover, in the intervention group, the changes in children’s depression and manifest anxiety scores significantly decreased; however, these changes were not significant in the control group.

The interpretation of the effect size was as follows: 0.0–0.19, trivial; 0.20–0.49, small; 0.50–0.79, moderate; and >0.80, large (19).

The results also show revealed that the total score of the strengths and difficulties questionnaire significantly changed in each group over time (p = 0.004). However, these changes were not significant in the two groups (table Table 5).

As seen displayed in table 6, parental Depression-Anxiety-Stress Scale score in each group did not significantly changed over time (p = 0.053). Moreover, this score was not significantly different in the two groups (P = 0.549).

## Discussion

This study aimed at examining the effectiveness and implementation of FRIENDS for Life as a preventive and therapeutic program for children with early or mild signs of anxiety or depression.

 The results of the present study revealed that children’s manifest depression and anxiety score before intervention was not significantly different in the experimental and control groups; however, their changes immediately after intervention and during 3 months of follow-up were significantly different with a large effect size (>0.80). This finding is consistent with the results of a study by Short (2001) on the effect of the Friends for Life program on the treatment of children with anxiety and their parents (13). Studies by Barrett (2001 and 2003) and Lowry-Webster also revealed a significant decrease in anxiety symptoms in 10 to 12 year-old children, which was consistent with the results of the present study (12, 34, 35).

The only study conducted in Iran was a research entitled “A Parent-only Group Cognitive Behavioral Intervention for Children with Anxiety Disorders: A Control Group Study”. This study was conducted by Shahrivar et all in 2009. The findings of their study showed no significant change in the total score of the Children’s Manifest Anxiety Scale (16). The reason might be that in their study the intervention employed parent session component of FRIENDS program as a psychoeducational model for parents of children with anxiety disorders, and training sessions were not held for the students. While in our study, training program was held for children themselves. Moreover, 2 training sessions were held between and at the end of the sessions for parents to increase the program’s effect, which seemed to have played a major role in reducing anxiety and depression in children.

Cooley-Strickland (2011) evaluated the efficacy of a school-based anxiety prevention program among urban children exposed to community violence. They divided 98 children aged 8 to 12 into 2 groups (intervention and waiting list). The intervention was performed for 13 sessions, twice a week using the modified version of the Friends for Life program. Anxiety symptoms significantly decreased in both groups. Moreover, reading achievement scores significantly increased in both groups. However, no significant difference was observed between the 2 groups in exposure to social violence, children’s manifest anxiety, academic performance, and life’s unfortunate events. Increased mathematics achievement scores, decreased life stressors, and reduced victimization by community violence were observed only in the intervention group (15). The results of that study are inconsistent with the results of the present study. The difference in results could be due to different study samples in the 2 studies, which were exposed to social violence. Moreover, cultural and racial differences could explain the lack of significant difference between the 2 groups in the study by Cooley-Strickland.

A study by Barrett, Farrell, Ollendick, and Dadds in 2006 revealed that 85% of the children who participated in the Friends for Life program and had clinical symptoms of anxiety and depression above the cut-off point were treated after 12 months. In the control group, this rate was 31%. This positive effect remained stable up to 3 years after participation in the Friends for Life program. This research confirmed the effect reported by Barrett and Luck in 2003 on decreased anxiety after the Friends for Life intervention program. It also revealed that the long-term effect of intervention could remain stable for 36 months (36). In the present study, in addition to anxiety symptoms, low mood symptoms were also investigated. Significant changes were observed in the intervention group. Therefore, the present study strongly confirms the positive effect of the Friends for Life program on improving low mood symptoms in highly anxious children. This positive effect remained stable during the 3- month follow-up. Other studies also found that implementation of the Friends for Life program could reduce anxiety and depression symptoms immediately after the end of the program, and it could be effective for 1 to 3 years (13, 36-39). Moreover, Barrett, Farrell, Ollendick, and Dadds conducted a study on 9 to 10, and 14 to 16 year-old children. Their comparison revealed that both groups enjoyed the Friends for Life program; however, the younger group showed higher reduction in anxiety symptoms (36, 40). The present study also investigated this age group and found positive results.

Stallard, et al. (2012) conducted an intervention at schools based on the protocol of the Friends for Life program to reduce anxiety in children. The intervention was conducted in 9 weekly sessions for 9 to 10 year-old children with anxiety and mood symptoms. Preliminary results and 6-month follow-up were indicative of reduction in anxiety and depression in children, and positive changes were made in self-esteem, concern, bullying behaviors, and life satisfaction in children (41). The results of the 12-month follow-up of the present study, which was published in 2014, showed that compared to the group receiving intervention from teachers, significant reduction in anxiety and depression scores was observed in the group receiving intervention from mental health professionals. However, no significant difference was found between the 2 groups in the evaluation tests completed by parents and teachers. These researchers suggested that universal programs for the prevention of anxiety can be effective when implemented at schools; however, their effectiveness will be different depending on who provides them (42). The results of the 24-month follow- up of their study, which was published in March 2014 by the same authors, also showed that the intervention results were still stable after 24 months (43). The trainer plays a significant role in the effectiveness of the program in reducing anxiety and depression. One of the strengths of our study was that the training was performed by a child and adolescent psychiatrist, which could increase the effect size. Application of Friends for life intervention in the school setting provides the opportunity to reach large numbers of children in a relatively safe and non-stigmatizing environment. By focusing on the 9 to 10 year age group, the unauthorized absences that tend to increase in the secondary school are minimized.

The results also revealed that the total score of the Strengths and Difficulties Questionnaire significantly changed in each group over time. However, these changes were not significant in the 2 groups. Most of the studies on the effectiveness of the Friends for Life program have not investigated the SDQ test. A study by Stallard et al. indicated that the SDQ test will be completed by parents in the next follow-up studies. The only similar study was that of Shahrivar in 2009. In that study, children’s anxiety behaviors, which were based on the emotional symptoms subscale score of Strengths and Difficulties Questionnaire and were scored by parents, significantly decreased. Moreover, the total score of this scale, which was reflective of children’s behavioral problems in different areas, decreased although this change was not statistically significant. In the study by Shahrivar et all, the samples receiving training included the parents of the children with anxiety disorder who received medications. However, in the present study, the children were trained, their anxiety symptoms were mild to moderate, and they did not receive medications. Another important finding of the study by Shahrivar et all was that although parental anxiety scores in the Depression Anxiety Stress Scale did not change, parental depression scores significantly decreased. Moreover, the total score of the depression anxiety stress scale decreased, which was not statistically significant (16). Although the group receiving training in the above-mentioned study was different from the one in the present study; the same results were obtained in the present study. Parental depression-anxiety-stress scale score did not change significantly in the 2 groups over time. Because the role of parental depression and anxiety as a predisposing factor for anxiety and depression in children is emphasized, it seems that more attention should be given to the treatment of such disorders in the parents of children with anxiety and depression.

Application of Friends for life intervention in the school setting provides the opportunity to reach large numbers of children in a relatively safe and non-stigmatizing environment. By focusing on the 9 to 10 year age group, the unauthorized absences that tend to increase in the secondary school are minimized.

## Limitations

Studies on children with severe anxiety and depression should be conducted and their results are recommended to be compared with those of the present study. Moreover, studies that evaluate the effectiveness of combined parent-child cognitive behavioral therapy in Iran will be useful. Conducting more studies in Iran on other age groups of children (preschoolers and adolescents) seems necessary.

Difficulties in implementation and intersectoral collaboration for such training programs at Iranian schools were among the limitations of this study. Moreover, because the current society considers psychological symptoms a label, the level of cooperation by training centers, children’s parents, and children themselves will be affected. However, as the principal and deputy principal of the given school were well aware of psychological issues and parents were entirely justified, this limitation was minimized. 

## Conclusion

The present study provides insight into the implementation and effectiveness of “FRIENDS for Life” program as a school-based preventive program for children with early or mild signs of anxiety or depression.
